# Fractionated Radiation Therapy for Large and Giant Cavernous Sinus Hemangioma: A Retrospective Study

**DOI:** 10.3389/fneur.2020.00355

**Published:** 2020-05-13

**Authors:** Zengfeng Xin, Yihan Yao, Guodi Chen, Liancong Wang, Meibao Shu, Qinghua Lv, Haifeng Yu, Ting Zhang

**Affiliations:** ^1^Department of Radiation Oncology, Zhejiang University School of Medicine, The Second Affiliated Hospital, Zhejiang University, Hangzhou, China; ^2^Department of Orthopedic Surgery, Zhejiang University School of Medicine, The Second Affiliated Hospital, Zhejiang University, Hangzhou, China; ^3^Key Laboratory of Tumor Microenvironment and Immune Therapy of Zhejiang Province, Hangzhou, China; ^4^Department of Chemotherapy Center, Zhejiang Cancer Hospital, Hangzhou, China

**Keywords:** hemangioma, cavernous sinus, central nervous system, radiotherapy, cognitive function

## Abstract

**Purpose:** Surgical resection has been traditionally used as a treatment for cavernous sinus hemangioma (CSH). However, this is usually difficult due to tumor vascularity and results in complications especially in large and giant CSH (volume >20 cm^3^). Previous studies have reported that radiotherapy (RT) provides an alternative treatment modality for hemangiomas. However, the optimized dose and fractions which control CSH and also protect the cognitive function remain unclear. This study reports our experience in the management of symptomatic large and giant CSH.

**Methods:** Fifty-four patients with symptomatic large (20 cm^3^ <tumor volume ≤ 40 cm^3^, 3–4 cm in diameter) and giant (tumor volume>40 cm^3^, >4 cm in diameter) CSH were enrolled in a retrospective study between January 2007 and December 2018. The prescription dose to the target margin was 50 Gy in 25 fractions.

**Results:** The mean pre-RT tumor volume was 60.9 cm^3^ which ranged from 20.2 to 230.5 cm^3^. The clinical data obtained was analyzed retrospectively following a mean follow-up period of 35.0 months which ranged from 1 to 140 months. All patients experienced tumor shrinkage within 3 months after radiotherapy. There was an average mean tumor reduction of 79.7% (range, 48.4–98.5%) with no patients experiencing tumor progression and recurrence. All the 54 patients experienced symptomatic improvement within 1 month to 12 months after radiotherapy. Within the entire follow up period, no patients experienced any form of permanent complications or symptomatic radiation toxicity. Neurocognitive impairment studies were conducted before and after radiotherapy on 28 patients while the studies were conducted after the last follow up in 40 patients. The cognitive function of all the participants had normal MoCA-scores of 28.25 pre-radiotherapy. The post-treatment MoCA-scores were also clinically stable (28.04, *p* = 0.78), and the average MoCA-score did not show any decline until the last follow-up (27.61, *p* = 0.13).

**Conclusion:** The optimal dose and fractions of radiotherapy treatment for symptomatic large and giant cavernous sinus hemangioma remain unclear. This study, therefore, used a marginal dose of 50 Gy in 25 fractions in radiotherapy and this was proven to be effective and relatively safe in the treatment of symptomatic large and giant CSHs.

## Introduction

Hemangioma is a benign vascular tumor which causes clinical symptoms due to its progressive tumor growth and large size (1). Cavernous sinus hemangioma (CSH) is a rare benign tumor arising from the cavernous sinus and constitutes <3% of sellar or para sellar tumors, and tend to occur mostly in middle-aged females (2). CSHs can develop with no elaborate symptoms in the cavernous sinus (CS). Nonetheless, large (20 cm^3^ <tumor volume ≤ 40 cm^3^,3–4 cm in diameter) and giant (tumor volume>40 cm^3^, >4 cm in diameter) CSHs may present with a variety of neurologic symptoms, such as blurred vision, diplopia, headache, seizures, and neurological deficit (3). The fatality rate in the case of a rupture or bleeding is extremely high.

For this type of benign tumor, treatment can be achieved through the complete removal of CSH. Currently, the available treatments for CSH include microsurgical resection, embolization, stereotactic radiosurgery (SRS) such as Gamma Knife radiosurgery (GKRS), and fractionated radiotherapy (4, 5). The resection of large and giant CSHs is challenging due to the complex natural anatomic structures around the tumor (6). Surgical management of this lesion is also associated with extremely high risks due to the possibilities of significant blood loss and cranial neuropathies, hence, it is difficult to achieve total resection. Despite the recent advances in neurosurgical techniques, the total resection rate of CSHs by surgical excision is 64%, and cranial neuropathies-related symptoms deteriorate after surgical treatment in ~71% of the patients (7, 8).

Similar to the high risks of surgical treatment, radiotherapy (RT) for CSHs management has been extensively studied and reported. For example, in 1999, a postoperative patient with a residual lesion after resection was treated using GKRS. The postoperative results showed that the tumor markedly decreased and the preoperative clinical symptoms improved (9). Radiosurgery is currently an optimal choice for primary or postoperative treatment of CSH since it has demonstrated favorable clinical results. However, radiosurgery such as GKRS is suitable for small lesions (10, 11). Yamamoto et al. described their experiences of the successful treatment of about 30 cases of CSHs using radiosurgery. In their study, the average tumor volume was 11.5 cm^3^ (12). Due to the high morbidity of radiation complications, the size and location of the tumor limit the usage of GKRS for large CSHs, especially those adjacent to critical anatomic structures. Large lesions, therefore, require a larger target area and this may also lead to critical complications during irradiation for example on normal structures such as cerebral tissue and important nerves. Therefore, the use of fractionated stereotactic irradiation is increasing. In a previous study, hypofractionated stereotactic radiotherapy (H-SRT) was proven to effectively reduce the volume of CSH without causing severe radiation complications, even in large tumors with a median tumor volume of 19.3 cm^3^ (the range was from 15.8 cm^3^ to 69.3 cm^3^) (13). In another study, 14 large cavernous sinus hemangioma (volume >20 cm^3^) patients were treated using H-SRT, and the reported reduction rate of the tumor volume was 77%, and with no radiotherapy-induced complications observed. These studies have confirmed the effectiveness and safety of H-SRT (14). The reduction in the damage of normal structures while decreasing the volume of neoplasms is the main biological benefit of fractionated RT (15). For the current study, to achieve favorable reduction as well as to protect the optic pathway and other normal tissue from irradiation, this study's regimen of RT was used to deal with large and giant CSHs. In this retrospective study, the experience of 54 patients with large and giant CSHs in our institution was described. Therefore, the aim of this study was to evaluate the efficacy and safety of our regimen of RT as a treatment modality for large and giant CSHs.

## Methods and Materials

### Patients

Patients diagnosed with large and giant CSHs (tumor volume>20 cm^3^) and treated with RT at the Second Affiliated Hospital of Zhejiang University from January 2007 to December 2018 were recruited in the study. There were 54 patients diagnosed during the stipulated period and their characteristics are presented in Table 1. This study was approved by the Ethics Committee of the Second Affiliated Hospital of Zhejiang University School of Medicine (No. 2019–220).

**Table 1:** Baseline demographic and clinical factors of patients with large and giant CSHs.

**Variable**	**Results**
Age at study media (range)	50.2 (22-66)
Age at diagnosis media (range)	47.8 (22-64)
Sex (Male: Female)	42:12
Length of follow up (month range)	35.0 (1-140)
Symptom	*N* (%)
Blurred vision	33 (61.1)
Headache	18 (33.3)
Vomit	6 (11.1)
Ptosis	3 (5.6)
Proptosis	5 (9.3)
Dizziness	21 (38.9)
Vertigo	4 (7.4)
Diplopia	3 (5.6)
Hemiplegia	1 (1.9)
Weakness of upper extremity	1 (1.9)
Diagnosis	*N* (%)
Biopsy	5 (9.3)
MRI+DSA	5 (9.3)
MRI	44 (81.4)
Tumor volume	
Pre-treatment (cm^3^) (range)	60.9 (20.2–230.5)
Post-treatment (cm^3^) (range)	10.3 (1.3–39.1)
Volume reduction (%) (range)	79.7 (48.4–98.5)
Symptom changes after RT	*N* (%)
Recovery	51 (94.4)
Improved	3 (5.6)
Stable	0 (0)
Dose/Fraction (Gy/F)	*N* (%)
50/25	45 (83.3)
46/23	5 (9.3)
40/20	2 (3.7)
45/15	2 (3.7)
ECOG	*N* (%)
0	22 (40.7)
1	25 (46.3)
2	4 (7.4)
3	2 (3.7)
4	1 (1.9)
Education level	*N* (%)
Completed College/University	14 (25.9)
Completed high school	20 (37.0)
Less than high school	15 (27.8)
No education	5 (7.3)

*Pt. No., patient number; M, Male; F, Female; RT, radiotherapy; MRI, Magnetic Resonance Imaging; DSA, Digital subtraction angiography*.

### Diagnosis

A brain magnetic resonance imaging (MRI) and/or computed tomography (CT) study was used as the primary diagnostic tool in all the patients. CSHs were mainly diagnosed based on some specific imaging characteristics using MRI where the CSHs had well-defined boundaries in the MRI images (16). On T1-weighted images, the tumor was uniformly hypointense compared with the brain parenchyma, and also showed significantly brighter hyperintensity on T2-weighted images. The lesions became significantly enhanced after a contrast agent injection (17). The diagnosis was confirmed through pathological diagnosis or typical neuroimaging diagnosis. In this study, a total of 5 patients were pathologically diagnosed, while 49 patients were diagnosed through MRI with or without digital subtraction angiography (DSA).

### Treatment Strategy

The Eclipse treatment planning system was used to fuse the CT and MRI scan with enhanced T1-weighted sequence to improve the target identification. The gross tumor volume (GTV) was defined as the enhanced tumor area according to the images. Expansion of the GTV margin by 5 mm in all directions was used to obtain the planning target volume (PTV). As shown in Table 2, evaluation of the target coverage, dose heterogeneity, and conformity were used to assess the quality of the treatment plans. 6-MV X-ray was used to deliver a total dose of 50 Gy in 25 fractions in 45 patients. However, 5 patients received 46 Gy in 23 fractions, whereas 2 patients received 40 Gy in 20 fractions and 1 patient received 45 Gy in 15 fractions. Plan normalized at 100% prescription cover 95% target volume. RT was performed using intensity modulated three-dimensional treatment plan across several different non-coplanar fixed fields in a linear accelerator (Varian).

**Table 2:** Treatment characteristics and dose volume analyses of 54 patients.

**Pt. No**.	**Maximum tumor dose (Gy)**	**Minimum tumor dose (Gy)**	**Average tumor dose (Gy)**	**Prescription tumor dose (Gy/F)**	**CI**	**HI**	**Coverage**	**Maximum optic nerve dose (Gy)**	**Maximum optic chiasm dose (Gy)**	**Maximum brainstem dose (Gy)**
1	57.25	47.96	52.67	50/25	0.82	1.13	94.7	43.41	51.49	39.12
2	52.88	48.11	51.57	50/25	0.77	1.06	92.1	21.03	21.18	25.11
3	56.09	48.83	53.72	52/26	0.77	1.07	97.1	21.63	47.39	49.90
4	57.69	48.12	53.91	52/26	0.83	1.09	93.9	44.12	52.13	50.12
5	55.43	47.98	52.16	50/25	0.81	1.10	94.3	43.17	51.78	49.93
6	54.04	43.25	51.43	50/25	0.79	1.08	93.8	52.53	52.08	52.01
7	50.39	43.76	47.85	46/23	0.78	1.09	93.7	48.67	48.24	47.79
8	46.89	38.21	43.17	40/20	0.76	1.12	92.6	42.78	43.76	43.42
9	53.58	47.72	51.46	50/25	0.83	1.07	94.0	48.15	43.32	47.59
10	49.76	42.55	46.75	45/15	0.76	1.11	91.7	47.23	44.84	46.19
11	55.39	41.68	51.86	50/25	0.79	1.09	95.2	46.37	42.22	40.69
12	50.23	40.21	46.21	44/22	0.81	1.08	94.2	40.12	37.54	38.76
13	55.25	47.66	53.62	50/25	0.84	1.12	95.2	44.41	51.12	41.14
14	46.97	41.91	46.01	45/25	0.81	1.04	95.5	46.65	46.97	46.21
15	49.65	37.60	46.42	45/25	0.80	1.10	95.0	46.30	46.69	47.18
16	55.04	43.97	51.99	50/25	0.86	1.09	94.6	42.19	50.66	29.41
17	54.11	46.80	51.37	50/25	0.88	1.07	95.3	52.60	51.93	50.88
18	54.95	45.20	52.03	50/25	0.78	1.10	95.6	53.00	53.62	53.40
19	55.25	47.93	51.59	50/25	0.87	1.08	94.5	42.12	49.23	35.97
20	53.88	48.91	50.87	50/25	0.87	1.09	92.4	25.76	50.53	28.43
21	55.09	48.97	53.53	50/25	0.79	1.02	97.8	31.33	46.69	44.90
22	56.73	49.15	51.17	50/25	0.84	1.09	95.2	44.12	40.83	45.65
23	52.78	48.08	51.96	50/25	0.83	1.11	94.4	42.87	50.68	49.44
24	53.14	45.45	51.68	50/25	0.75	1.04	94.8	52.12	51.48	51.33
25	51.49	44.32	48.21	50/25	0.79	1.12	94.7	45.67	47.32	48.23
26	53.67	46.21	49.17	50/25	0.78	1.08	94.6	42.45	44.74	42.68
27	54.15	47.72	51.96	50/25	0.84	1.11	94.4	48.21	45.39	45.21
28	55.99	47.55	49.98	50/25	0.86	1.09	94.7	46.11	43.23	43.13
29	54.97	41.68	51.86	50/25	0.82	1.09	95.1	43.37	42.52	46.69
30	51.04	46.21	49.92	50/25	0.84	1.05	94.6	41.14	34.54	35.56
31	54.89	47.69	53.52	50/25	0.85	1.11	95.3	43.41	49.42	40.74
32	47.65	41.91	47.01	45/25	0.83	1.07	95.4	44.65	45.97	46.11
33	48.89	42.60	47.42	45/25	0.81	1.11	95.1	44.30	46.59	46.18
34	55.32	45.97	51.43	50/25	0.87	1.08	94.7	42.21	45.66	34.41
35	53.96	47.80	51.57	50/25	0.88	1.09	95.4	52.70	52.93	52.85
36	55.02	46.20	52.07	50/25	0.79	1.11	95.5	52.00	53.62	52.58
37	56.91	47.56	52.37	50/25	0.84	1.12	94.6	43.41	46.49	41.12
38	53.12	47.54	51.47	50/25	0.79	1.07	94.1	26.73	50.08	28.11
39	55.49	48.89	52.79	52/26	0.78	1.08	96.1	20.63	44.39	48.90
40	58.09	48.78	53.21	50/25	0.84	1.08	94.9	46.12	50.13	51.12
41	54.97	46.32	50.16	50/25	0.82	1.11	94.4	46.17	50.78	49.33
42	53.46	47.25	51.93	50/25	0.89	1.08	94.8	51.53	51.08	51.01
43	51.37	46.76	48.85	46/23	0.79	1.09	94.7	49.67	45.24	47.89
44	49.68	42.21	47.17	46/23	0.78	1.11	93.6	40.78	41.76	43.48
45	53.68	48.92	51.87	50/25	0.84	1.08	94.1	47.15	43.23	44.59
46	50.96	43.55	47.75	46/23	0.78	1.10	92.7	45.23	43.84	47.99
47	53.54	42.68	51.86	50/25	0.76	1.04	95.6	46.37	47.22	43.69
48	49.12	43.21	47.21	46/23	0.88	1.07	94.3	41.12	35.54	33.76
49	54.89	48.66	53.62	50/25	0.85	1.11	95.3	43.41	50.42	40.13
50	48.34	43.92	47.03	45/25	0.82	1.05	95.4	43.65	46.57	42.21
51	53.95	46.60	51.42	50/25	0.81	1.11	94.6	43.30	43.69	46.18
52	55.32	46.97	51.34	50/25	0.87	1.09	95.6	42.11	44.66	32.41
53	54.55	47.80	51.41	50/25	0.87	1.08	95.4	53.60	52.93	51.88
54	54.47	46.20	52.76	50/25	0.79	1.08	95.5	53.54	53.65	53.67

*Pt. No., patient number; CI, conformity index; HI, homogeneity index*.

### Neurocognitive Function

The Montreal Cognitive Assessment (MoCA) was used to assess the neurocognitive performance of the patients (18). This assessment results were presented in the form of a total score and seven scores in seven different aspects: naming, memory, attention, language, executive and visuospatial functioning, abstraction and orientation (score 0–30). In this study, a score of <26 was used to define neurocognitive impairment (NCI) (19).

### Follow Up Period

The first follow-up was conducted 3 months after the completion of RT, and subsequent follow-ups were conducted annually after the treatment. During each follow-up, a questionnaire was administered to obtain the patients clinical status and any other relevant physical examination, as well as brain MRI or CT scan, were performed. Based on MRI or CT results, the Eclipse planning system (version 13.6, Varian Medical Systems, Palo Alto, CA, USA) was used to estimate the tumor volumes. The National Cancer Institute Common Terminology Criteria for Adverse Events version 4.0. was used to score and document all treatment-induced toxicities.

### Statistical Analysis

Basic descriptive statistics were used to demonstrate the baseline characteristics of patients, treatment information, and other variables measured during treatment and the follow-up period. For binary results, univariable analyses were conducted using a logistic regression model whereas linear regression was used for continuous variables*. t*-tests were used to analyze the difference between pre-treatment mean MoCA scores and post-treatment mean MoCA scores. All the statistical analyses were performed using SPSS software, version 20.0.

## Results

### Patients Characteristics and Treatment Modalities

A total of 54 patients with CSH and treated between January 2007 and December 2018 were included in the study. As shown in Table 1, the majority of the patients were female (77.8%, 42) with a median age of 47.8 years old (age range was from 22 to 64 years) at diagnosis. Prior to RT, five patients received operations, 4 of them underwent lesion biopsy while another one received partial resection. The most common initial symptoms among the patients were blurred vision (61.1%, 33), dizziness (38.9%, 21), headache (33.3%, 18), vomit (11.1%, 6), proptosis (9.3%, 5), ptosis (5.6%, 3), vertigo (7.4%, 4), and weakness in upper extremities (1.9%, 1). The mean hemangioma volume was 61.3 cm^3^ (the range was between 20.2 to 230.5 cm^3^) before treatment.

**Figure 1:**
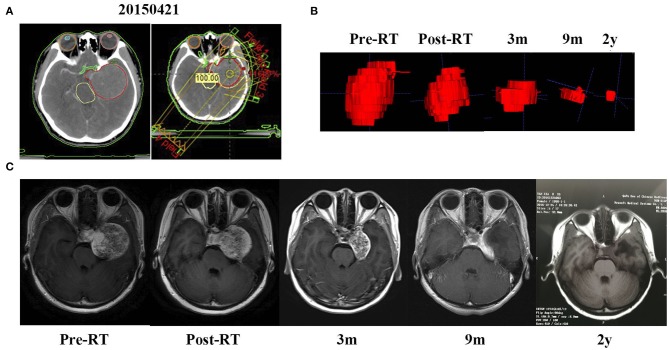
A mass occupying the left cavernous sinus of a 24-year-old woman (patient 1) was shrunk gradually after RT. **(A)** Treatment plan based on CT scan. **(B)** The 3D imaging of the mass before RT and at a different time of follow up after RT. **(C)** MRI scan before RT, the day after RT completion and 3 months, 9 months, and 2 years after RT.

Treatment characteristics of each patient are presented in Table 2. The RT was performed at the median 95% isodose line. The mean tumor coverage receiving the entire prescribed dose was 94.7% (ranging from 91.7 to 97.8%), and the mean conformity index was 0.82 (ranging from 0.76 to 0.88). In addition, the average tumor maximum dose was 53.36 Gy (ranging from 46.97 to 58.09 Gy), while the mean minimal dose of the tumor was 45.74 Gy (ranging from 37.60 to 49.15 Gy). Furthermore, the average maximum dose irradiating to the ipsilateral optic nerve, optic chiasm, and brainstem were 43.58Gy (range was between 21.03 and 53.60Gy), 46.67 Gy (range was between 21.18 and 53.62 Gy), and 44.19 Gy (range was between 25.11 and 53.67 Gy), respectively. Figure 1 shows one of the treatment plans.

### Imaging Response

**Figure 2:**
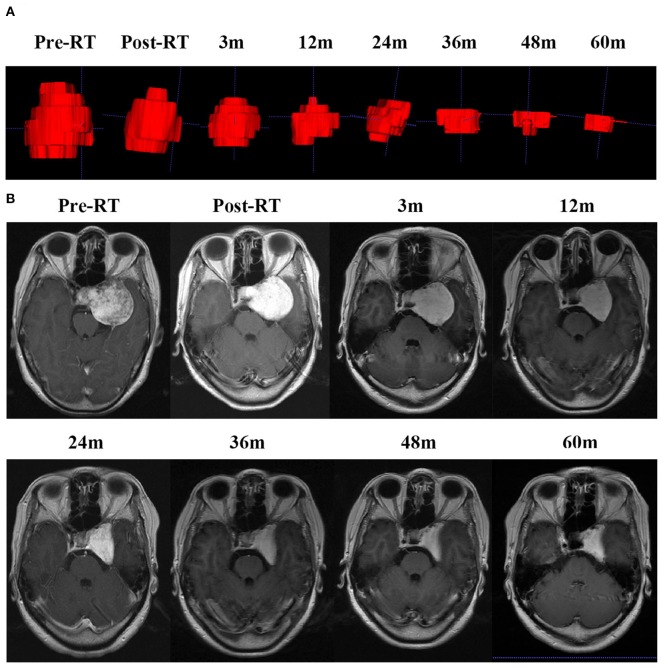
CSH of patient 7 kept decreasing even 5 years later. **(A)** The 3D imaging of the mass before RT and at different times of follow up after RT. **(B)** MRI scan before RT, the day after RT completion and at a different time of follow up after RT.

**Figure 3:**
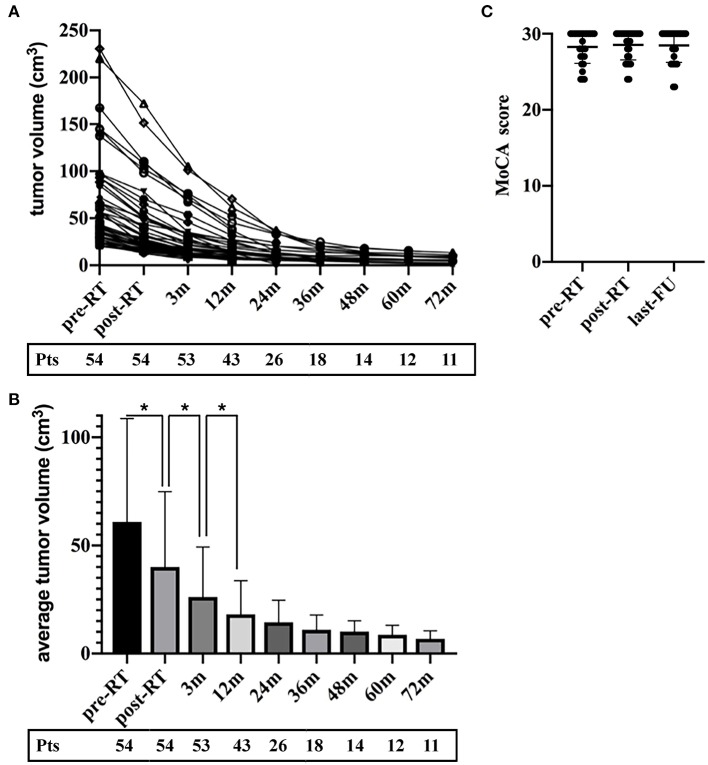
The tumor volume reduced over the entire follow-up period without transient enlargement in all the patients. **(A)** The volume of the CSHs decreased in all patients. **(B)** The percentage of tumor volume during the follow-up intervals to their initial volume. **(C)** The MoCA score of participants at three-time points (pre-treatment, post-treatment and the last follow-up).

The average follow-up period for all the patients was 35.0 months (ranging from 1 to 140 months). The MRI or CT images demonstrated significant tumor shrinkage in all the patients and the tumors control rate was 100%. Shrinkage of the CSHs was seen immediately after the completion of RT. The tumor volume was about 65.7% of their initial volume at the end of the RT and kept decreasing during the follow-up period. Three months post RT, the masses had reduced in size to 42.9% of the original volume (Figures 3A,B). At 1-year post RT, they reduced further to 29.7% and 23.6% at 2 years post-RT. In some patients, the tumor sizes remained stable without any change in size while on others, they kept decreasing even after 5 years follow-up (Figures 2A,B). The MRI results indicated a 98.7% tumor volume reduction 2 years after RT (Figure 1). However, no transient tumor enlargement was observed in participants after RT.

### Clinical Response

All the patients had favorable neurological outcomes after the treatment. The neurologic symptoms changes after treatment are shown in Table 1. All pretreatment symptoms had recovered or improved within 1 month to 1 year after RT. Fifty-one patients (94.4%) experienced symptoms recovery after the treatment, while three patients (5.6%) had their symptoms improved which lasted for many years. During the final follow-up, all the participating patients showed favorable RT neurological outcomes. Only one patient expressed feelings of dizziness.

### Toxicity

There was no patient who suffered severe acute or delayed complications associated with RT. All the observed side effects were mild and transient. Grade 1 nausea and anorexia developed in only two patients (3.7%). While Grade 2 nausea occurred in three patients (5.6%) during the course of RT. However, these symptoms of RT-related side effects were managed with corticosteroids and they completely disappeared. MRI showed that some patients had encephaledema about 2 years after RT, but they had no clinical symptoms. There were no other acute or early delayed side effects from RT observed both during the treatment and follow-up period.

### Neurocognitive Assessment

Among 54 participating patients, 40 patients had the neurocognitive test done and they completed the MoCA at least once. Among them, 28 patients had the neurocognitive impairment study before and after RT while 40 patients did the study during the last follow-up. The cognitive functions of the participants were in the normal range (total MoCA-scores of 28.25) pre-RT. In comparison, post-treatment MoCA-scores were clinically stable (28.04, *p* = 0.78) and the average MoCA-score was not decreased at the last follow-up (27.61, *p* = 0.13) (Figure 3C).

## Discussion

In this retrospective study, the efficacy of radiotherapy as a treatment modality for patients with large and giant CSHs was evaluated. RT of a marginal dose of 50 Gy in 25 fractions at the median 95% prescription isodose line was used. The results of this study demonstrated an average 79.7% tumor reduction. Moreover, the pretreatment symptoms such as visual disturbance and headache could be cured or improve after RT. In all the patients there was no severe radiotherapy-induced complications observed during the treatment and neither during the subsequent follow-up period. The cognitive function of the patients did not also change during the entire treatment and follow-up period. These results revealed the efficacy and safety of our regimen of RT for large and giant CSHs.

Surgery is a traditional treatment for CSHs, however, in spite of there being several advanced surgical techniques, it is difficult to completely remove large and giant CSHs through surgery. This is due to the complex and critical anatomic structures in the cavernous sinus and the possible risk of excessive bleeding during the operation. Previous studies have demonstrated a low total surgical removal rate and high morbidity of complications after surgery in CSH (1, 17). Majority of the cases reported arose from the neurosurgery department.

Radiotherapy has become an emerging approach for CHS treatment for patients with residual tumors after surgery as well as those with great intraoperative risk. This is due to its efficacy and safety compared with surgical treatment. As early as the 1980s, radiosurgery was used for the treatment of high surgical risk CSH and has been successfully used as adjuvant therapy in CSH patients having residual lesions after surgical removal (9, 20). Subsequently, more studies have been conducted on the function of radiotherapy for CSHs due to its remarkable tumor shrinkage effect. Gamma Knife surgery is one of the radiotherapy techniques which has been proven to be safe and is being used for primary as well as postoperative management of CSH patients. A retrospective study analyzing the effect of Gamma Knife surgery in patients with CSH reported an average tumor volume reduction of 82%, and most of the pretreatment symptoms had been improved (21). In another meta-analysis, by Wang et al. 59 patients with a mean follow-up period of 49.2 months (range between 6 and 156 months) was studied. The results supported stereotactic radiosurgery as an alternative treatment for CSHs because of its reduction in traditional surgery-associated complications (22). However, the treatment outcomes of GKRS were associated with the tumor volume, underdosed tumor volume and total treated volume (23). Single-shot radiosurgery was reported to be effective for patients with small- or middle-sized CSHs. The high risk of radiation-induced optic neuropathy (RION) limits the usage of single-shot stereotactic radiosurgery for the patients with large CSHs (23). However, for large and giant cavernous sinus hemangiomas, Gamma Knife radiosurgery may not the optimal treatment. Previous studies, indicate that the risk of radiation-induced complications such as cranial nerve palsies and cognitive damage, could be higher if fractionation is not performed (24). Wang X. reported hypofractionated stereotactic radiosurgery using CyberKnife with 21 Gy in 3 fractions or 22 Gy in 4 fractions for the treatment of giant CSHs (volume>40 cm^3^, >4 cm in diameter) in 31 patients. Compared with pretreatment tumor volume, the results demonstrated a median tumor volume reduction of 88.1% (62.3–99.4%) during the last follow-up (3), with a mean follow-up period of 30 months. However, there was a concern about cognitive impairment as a long-term side effect.

It has been reported that cognitive impairment is one of the radiation encephalopathies which may significantly affect a patient's quality of life (25, 26). Radiation-induced cognitive impairment can be divided as acute, early delayed and late effects, with late effects being considered to be irreversible (27). The mechanism of the effect of RT on cognitive function has been reported in previous studies (25). Radiotherapy induces vascular damage and changes in white matter, including demyelination and coagulative necrosis (28). One of the essential regions of cognition is the hippocampus which has been found to be damaged after radiation. In some preclinical studies, neurogenesis inhibition following brain radiotherapy was demonstrated which caused cognitive impairment (29, 30). In this study, we followed up on the long-term effect of RT, however, no cognitive impairment was observed.

Fractionated stereotactic radiotherapy is another alternative treatment modality for large and giant CSHs especially those showing great risk through traditional surgery and radiosurgery. However, studies about conventional radiotherapy performed for CSH are rare with most of those available being case reports. In these relevant studies, the radiation dose varied from 30 Gy to 50 Gy (31). A phase II study focusing on hypofractionated stereotactic radiotherapy for large (volume> 20 cm^3^) CSH reported a mean of 77% tumor volume reduction (range, 44–99%), 14 patients included, and the radiotherapy dose was 21 Gy delivered in 3 fractions, however the mean follow-up period was 15 months (range, 6–36 months) (14). The current study focused on large and giant CSHs and has a longer follow-up period. As a benign tumor, long-term survival is expected, and it is necessary to minimize the toxicity of RT such as RION (32, 33).

This study, however, had a number of limitations. First, it was a retrospective and single-centered study with a small number of patients to make a definitive conclusion. Therefore, this needs further research and the inclusion of larger sample size in a multicenter study. Besides, for observing long-term cognitive deficits, the follow-up time period was relatively short, longer and more extensive follow-up is needed. The assessment of neurocognitive function involves many factors which have an effect on the MoCA scores, therefore, it was difficult to balance the factors due to the limited sample size. In addition, MoCA score is not the optimal tool for neurocognitive assessment in brain tumor patients. Furthermore, this study did not include a control group, and both the patients and investigators were aware of the treatment, which may exert influence on symptom reporting. Overall, further research on radiotherapy on CSH is required.

## Conclusions

MRI helps clinicians to make a diagnosis in large and giant cavernous sinus hemangioma, but the optimal treatment for symptomatic CSHs remain unclear. Although longer follow-up is needed, RT using a marginal dose of 50 Gy in 25 fractions was found to be effective and relatively safe in the treatment of symptomatic large and giant CSHs.

## Data Availability Statement

The datasets used and analyzed during the current study are available from the corresponding author on reasonable request.

## Ethics Statement

This study was approved by the Ethics Committee of the Second Affiliated Hospital of Zhejiang University School of Medicine (No. 2019–220).

## Author Contributions

TZ and YY designed this study and wrote the manuscript. GC, LW, and MS analyzed the clinical data and participated in the manuscript writing. TZ, YY, and GC interpreted the clinical data. HY and QL read and interpreted the radiology images. YY and LW collected the clinical data of surgery and radiotherapy. ZX and YY conceived the study and wrote the manuscript. All authors had read and approved the final manuscript.

## Conflict of Interest

The authors declare that the research was conducted in the absence of any commercial or financial relationships that could be construed as a potential conflict of interest.
